# Graph Neural Network for Protein–Protein Interaction Prediction: A Comparative Study

**DOI:** 10.3390/molecules27186135

**Published:** 2022-09-19

**Authors:** Hang Zhou, Weikun Wang, Jiayun Jin, Zengwei Zheng, Binbin Zhou

**Affiliations:** 1 School of Computer and Computing Science, Zhejiang University City College, Hangzhou 310015, China; 2 College of Computer Science and Technology, Zhejiang University, Hangzhou 310007, China; 3 Industry Brain Institute, Zhejiang University City College, Hangzhou 310015, China

**Keywords:** graph neural networks, protein–protein interaction, neural networks

## Abstract

Proteins are the fundamental biological macromolecules which underline practically all biological activities. Protein–protein interactions (PPIs), as they are known, are how proteins interact with other proteins in their environment to perform biological functions. Understanding PPIs reveals how cells behave and operate, such as the antigen recognition and signal transduction in the immune system. In the past decades, many computational methods have been developed to predict PPIs automatically, requiring less time and resources than experimental techniques. In this paper, we present a comparative study of various graph neural networks for protein–protein interaction prediction. Five network models are analyzed and compared, including neural networks (NN), graph convolutional neural networks (GCN), graph attention networks (GAT), hyperbolic neural networks (HNN), and hyperbolic graph convolutions (HGCN). By utilizing the protein sequence information, all of these models can predict the interaction between proteins. Fourteen PPI datasets are extracted and utilized to compare the prediction performance of all these methods. The experimental results show that hyperbolic graph neural networks tend to have a better performance than the other methods on the protein-related datasets.

## 1. Introduction

Proteins are versatile organic macromolecules that carry out a plethora of essential tasks for organisms. According to [1], almost 80% of proteins interact with some other proteins while performing their functions. There are some examples of biological processes that entail protein interactions, including DNA transcription and replication, hormone regulation, metabolism, antigen recognition, and signal transduction [2,3]. These interactions, which are known as protein–protein interactions (PPIs), are generated when two or more protein molecules come into physical contact. It is necessary to study PPIs and their various interaction types in order to obtain an in-depth understanding of the cellular biological activities in normal and diseased states, such as the antigen recognition and signal transduction in the immune system, which would provide insights into the exploration of molecular mechanism and protein functions [4]. These studies make it easier to identify therapeutic targets, synthesize drugs, and develop novel drugs [5,6].

In the past decades, there have been some high-throughput experimental methods proposed to detect the interaction between proteins, including the yeast two-hybrid screens (Y2H) [7], tandem affinity purification (TAP) [8], mass spectrometric protein complex identification (MS-PCI) [9], tandem affinity ourification and mass spectrometry (TAP-MS) [10], and affinity chromatography and co-immunoprecipitation (Co-IP) [11]. These experimental techniques can help build PPI databases for various species. However, these techniques have some shortcomings. The functional annotation of PPIs are updated slowly. In addition, the PPI data gathered by these techniques have a significant probability of false positives and false negatives due to the impact by the experimental setting and device resolution [12,13]. In addition, the cost is another major disadvantage of experimental methods because these experiments are time-consuming and labor expensive. More significantly, when a PPI can be detected through one single piece of experiment, it is hard to be fully interpreted [14]. Thus, reliable computational methods are required to learn knowledge from PPI data for a PPI prediction.

Recently, some computational methods have been developed in this field. The computational techniques can enhance the precision of PPI prediction when combined with experimental techniques [15,16]. These high-throughput computing techniques may be classified into two categories: traditional machine learning methods and deep learning methods. For the first category, several machine learning models were used to predict PPIs in order to increase the efficiency and accuracy, including Decision Trees [17], Naive Bayes [18], Random Forest [19], and Support Vector Machine (SVM) [20,21]. These machine learning methods seek to summarize entire sequence data pertinent to PPIs and quantify the properties of the 20 canonical amino acids. Deep learning techniques have an advantage over traditional machine learning techniques in that they can directly extract features from data and identify nonlinear relationships between extracted and learned features. They are capable to fully make use of the growing large-scale, high-dimension, and complex PPI datasets. Thus, deep learning techniques have gained extraordinary popularity in the past few years and have been effectively used to solve a variety of issues, including PPI prediction [22,23,24,25,26,27]. For example, Lei et al. proposed an effective computational technique based on a Multi-Modal Deep Polynomial Network for PPI prediction while embedding various data from protein properties [28]. Hashemifar et al. proposed a sequence-based convolution-based model to predict interactions between proteins and proteins [29]. Wu et al. proposed a recurrent neural network-based approach for neoantigen prediction, considering interactions between peptide–HLA (human leukocyte antigen) [30]. However, most of the previous deep learning-based methods only took into account sequence data, with a lack attention on the network data, e.g., the relationship with neighboring nodes, which has been shown to be useful for PPI prediction.

Graph neural networks have advanced significantly in the past few years and have become an important tool in graph-based studies, such as chemical stability prediction [31], the prediction of protein solubility [32], the modeling of polypharmacy side effects [33], and drug–target interactions [34]. For instance, Huang et al. employed a graph convolution model to predict the relationships between miRNA and drug resistance [35]. It has become popular to apply a graph neural network-based model for PPI prediction [36,37,38,39]. Yang et al. have presented a signed variational graph auto-encoder (S-VGAE) for protein–protein interactions prediction by treating PPI networks as indirect graphs, which can successfully utilize the graph structure and protein sequence information simultaneously [37]. Paradesi et al. exploited several structural features for the Saccharomyces cerevisiae PPI networks and utilized these features to train classifiers for the new protein–protein interaction prediction [40]. Song et al. presented an end-to-end model for predicting protein–protein interactions, with the utilization of predicted protein structural information [41]. By exploiting the topological data of PPI networks, You et al. devised a robust manifold embedding approach for evaluating the reliability of protein–protein interactions and forecasting new interactions [42]. Currently, most of the previous studies mainly investigate the applicability and feasibility of graph neural networks for protein–protein interactions prediction while learning graphical molecular representations of the proteins.

In this paper, we conduct a thorough comparison investigation of several graph neural network-based methods for protein–protein interactions prediction to evaluate the predictive capability of various methods. Five frequently used methods, including neural networks [43], graph convolutional neural networks (GCN) [44], graph attention networks (GAT) [45], hyperbolic neural networks (HNN) [46], and hyperbolic graph convolutions (HGCN) [47], are carefully contrasted using fourteen PPI networks datasets to look at the benefits and drawbacks of various approaches. These fourteen PPI networks datasets are extracted from the String database with seven types of interactions. The experimental results demonstrate that graph neural network-based models are powerful for solving the protein–protein interactions prediction tasks, and hyperbolic graph neural networks tend to have a better performance than the other methods on the protein-related datasets. The results of this study should serve as guidance and a point of reference for choosing appropriate graph neural networks to create predictive models for protein–protein interactions prediction.

The rest of this paper is organized as follows. Section 2 introduces the dataset used and the graph neural networks studied in this paper. Section 3 presents the comparative studies of different neural networks models. Finally, Section 4 concludes by summarizing the findings and drawing conclusions.

## 2. Materials and Methods

### 2.1. Datasets

#### 2.1.1. Data Collection

For the purpose of evaluating various graph neural networks, we draw upon the multi-type PPI data from the STRING database2 [48]. The STRING database is widely recognized as a reliable resource for research studies that are related to proteins. This database has compiled a comprehensive and objective global protein–protein interaction network by collecting, scoring, and integrating the information from the majority of publicly accessible sources of protein–protein interactions information, including direct and indirect interactions. Here, *direct* refers to physical interactions between proteins, while *indirect* refers to functional interactions between proteins.

#### 2.1.2. Data Preprocessing

In this study, the multiple interaction types between proteins using STRING is the main topic. PPI is divided into seven distinct categories, including reaction [49], binding [50], post-translational modifications (ptm) [51,52], activation [53], inhibition [54], catalysis [55], and expression [56]. At least one of these interaction types is present in every pair of proteins that interact with one another. Chen et al. generated two database subsets, extracting from the Homo sapiens subset of STRING database, by randomly selecting 1690 and 5189 proteins that match 40% of sequence similarity [57]. These two subsets are referred to as SHS27k and SHS148k, and they include 7624 and 44,488 multi-label protein–protein interactions, respectively.

**Figure 1:**
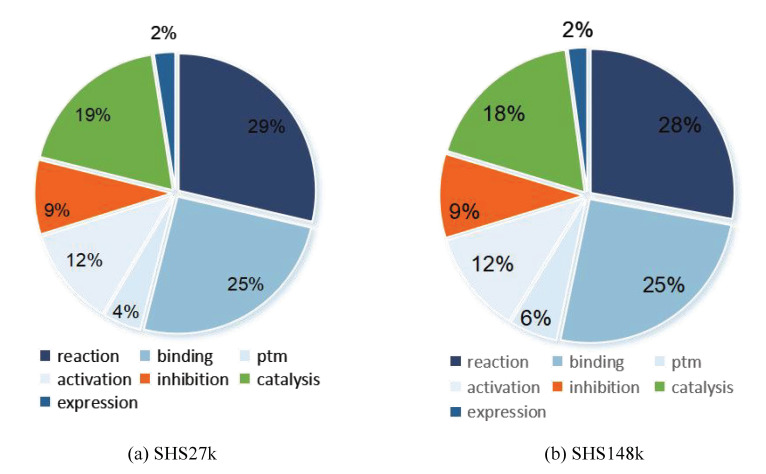
Interaction type distribution in two subsets.

On the basis of these two datasets, we extract each interaction between proteins and generate fourteen new datasets. In addition, we calculate the proportion of positive samples in these datasets, as shown in Table 1. From the table, we observe that the data-imbalance issue exists in the PPI datasets. Each dataset focuses on one unique protein–protein interaction. We also present two Pie figures to demonstrate the protein–protein interaction type distribution in the two datasets, as shown in Figure 1. From the two figures, we can observe that reaction accounts for the largest part in all the interactions. The interaction binding has obtained a comparable portion which indicates its importance in the protein functionalities. In the following section, we adopt various graph neural network-based models on these PPT datasets to evaluate the learning power and feasibility of graph neural networks on PPI.

### 2.2. Models

#### 2.2.1. Background

**Figure 2:**
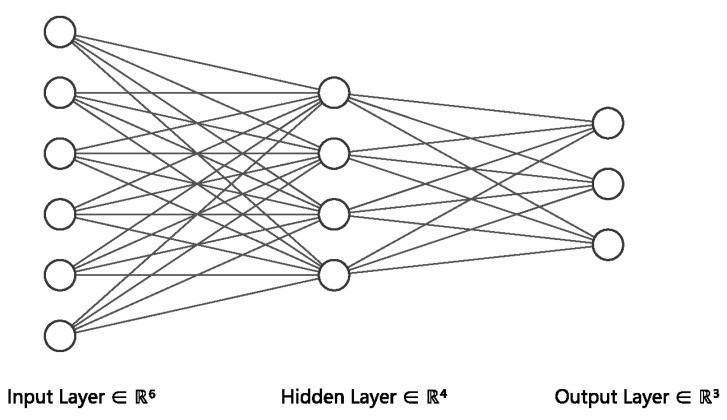
Neural Networks.

In this section, we introduce the background model, neural network, which is the key component of most graph neural networks. It is an algorithm inspired by the structure of human brain. Just like the human brain, it is an information response network topology in which many neurons process information and transmit information through the connections between neurons. For neural networks, we divide neurons into three layers: input layer, hidden layer, and output layer. Figure 2 shows the simplest three-layer neural network.

The input layer is the data that you need to process, and it will transfer the information to the hidden layer for processing. Each unit of the hidden layer contains an activation function, which multiplies the information transmitted from the input layer by different weights and then activates it, then transmits the processed information to the output layer. The unit of the output layer multiplies the information transmitted from the hidden layer by the corresponding weights to obtain your desired result. It should be noted that the whole network has only one input layer and one output layer, while the number of hidden layers can be determined according to the problem. In addition, there are several commonly used activation functions:

ReLU. It is the most commonly used activation function because of its simple implementation and good performance. Given the element *x*, the ReLU function is defined as shown in Equation (1).

(1) ReLU(x)=max(x,0)

Sigmoid. We often use gradient method to optimize the network, so we use sigmoid function as an approximation of a smooth and differentiable threshold unit, which transforms the input into the output on the interval (0, 1):

(2) $$\mathrm{sigmoid}\left(x\right)=\frac{1}{1+\exp(-x)}$$

Tanh. Similar to sigmoid function, tanh function can compress its input into the interval (−1, 1):

(3) $$\tanh\left(x\right)=\frac{1-\exp(-2x)}{1+\exp(-2x)}$$

#### 2.2.2. Graph Convolutional Neural Networks (GCN)

**Figure 3:**
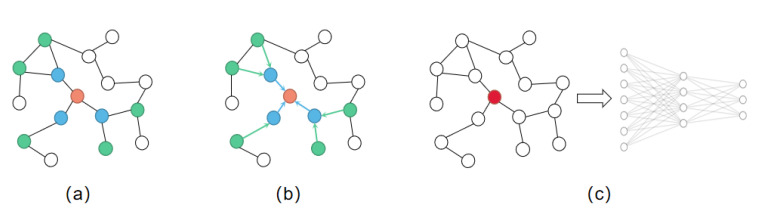
Graph convolution neural network. (**a**) Sample neighborhood. (**b**) Aggregate features from neighbors. (**c**) Taking the aggregated node features as the input of the neural network.

When dealing with the information of graph structure, neural networks can only input each element of the graph separately but cannot introduce the structure information of the graph. Therefore, some scholars put forward graph neural network, aiming at extracting features from graphs and introducing the structural relationship of graphs into the model. Graph convolution neural network (GCN) [44] is a commonly used method, and it works as shown in Figure 3.

When we extract the structural information of a graph, we aggregate the neighbor features of nodes. For GCN, the steps during aggregating are as follows:

Step 1. Consider the influence of neighbor nodes on the current node:
  (4) aggregate(X)=AX
  X is the feature matrix of the node.
Step 2. At the same time, the node itself should be considered:
  (5) $$\mathrm{aggregate}\left(X\right)=\hat{A}X$$
  (6) $$\hat{A}=A+I$$
  A is the adjacency matrix of graph, I is identity matrix.
Step 3. Symmetrical normalization. If the degree difference between two adjacent nodes is large, the node with smaller degree will be distorted after each round of aggregation. To reduce this effect, we need symmetrical normalization:
  (7) $$\mathrm{aggregate}\left(X\right)={\hat{D}}^{-0.5}\hat{A}{\hat{D}}^{-0.5}X$$
  (8) $$\hat{D}=D+I$$
  D is degree matrix of graph.
Step 4. Multiply the aggregated features by parameters and activate them:
  (9) $$X^{\left(l+1\right)}=\sigma \left({\hat{D}}^{-0.5}\hat{A}{\hat{D}}^{-0.5}X^{\left(l\right)}W^{\left(l\right)}\right)$$
  $W^{\left(l\right)}$ is the learnable parameters of layer l, $X^{\left(l\right)}$ is the feature matrix of layer l, σ is a nonlinear activation function.

#### 2.2.3. Graph Attention Networks (GAT)

Graph attention networks (GAT) [45] are another graph neural network. In GCN, we use symmetric normalization to solve the problem of node distortion, while in GAT, we introduce attention mechanism, so that neural network can learn to decide the influence of each neighbor node on itself.

After obtaining the learnable linear features from the input layer, the next step is to calculate the weight matrix. Each element in the matrix represents the influence between nodes, called attention coefficient, which is calculated as follows:

(10) $$\alpha_{ij}=\frac{\exp(\mathrm{LeakyReLU}({\tilde{a}}^{T}[W{\vec{h}}_{i}\left|\right|W{\vec{h}}_{j}]))}{\sum_{k\in N_{i}}\exp\left(\mathrm{LeakyReLU}({\tilde{a}}^{T}[W{\vec{h}}_{i}\left|\right|W{\vec{h}}_{k}])\right)}$$

$N_{i}$ is the set of all neighbor nodes of i node, LeakyReLU is an activation function, ${ã}^{T}$ is a learnable vector, W is a learnable matrix, $\vec{h}$ is the feature of node. "||" means to spell two vectors together. We can learn that the numerator is the influence of j node on i and the denominator is the influence of all neighbors of i on i. Our attention coefficient is the ratio of the two, which is also a normalization operation.

After calculating the attention coefficient of all the neighbors of node i and its own attention coefficient, we can calculate the new features of node i according to Equation (11).

(11) $${\vec{h}}_{i}^{{}^{\prime }}=\sigma \left(\sum_{j\in N_{i}}\alpha_{ij}W{\vec{h}}_{j}\right)$$

**Figure 4:**
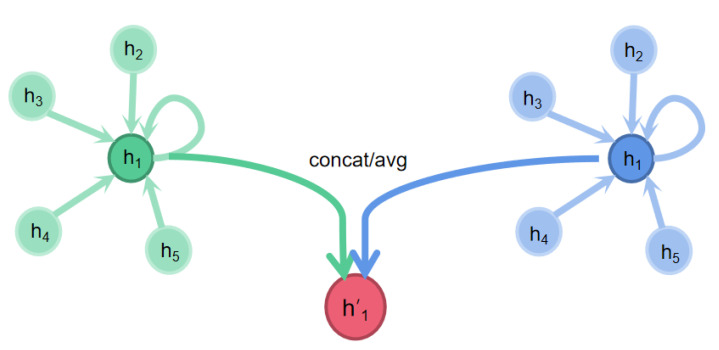
Multi-headed self-attention.

At the same time, it is worth noting that the data of graph structure have several independent features, and the feature $\vec{h}$ we input each time is only one of them. In order to ensure the effectiveness of attention mechanism, we need to introduce all independent features, respectively, which is called multi-head attention mechanism, as shown in Figure 4.

After the introduction of multi-head attention mechanism, the calculation formula of features is changed, as shown in Equation (12).

(12) $${\vec{h}}_{i}^{{}^{\prime }}=\overset{K}{\underset{k=1}{\|}}\sigma \left(\sum_{j\in N_{i}}{\alpha_{ij}}^{k}W^{k}{\vec{h}}_{j}\right)$$

*K* represents *K*-attention mechanism.

#### 2.2.4. Hyperbolic Neural Networks (HNN)

Hyperbolic neural networks (HNN) [46] generalize deep neural models to non-Euclidean domains by constructing hyperbolic geometric spaces, which can be used to visualize large categorical data or embed complex neural networks using their high capacity and tree-like nature, while performing far better than Euclidean spaces on hierarchical classification or inherited data. For HNN, important parts include multinomial logistic regression (MLR), feed-forward (FFNN), simple and gated (GRU) recurrent neural networks (RNN), and the steps during aggregating are as follows:

Step 1. According to the Möbius addition, the exponential mapping, the logarithmic mapping, the isometric method of parallel transport of the Levi-Civita connection, etc., it is possible to obtain a mapping relation from a vector v in a manifold to another tangent space $T_{x}D_{c}^{n}$, v∈($T_{x}D_{c}^{n}$), as shown in Equation (13).
  (13) $$P_{0\to x}^{c}\left(v\right)=log_{x}^{c}\left(x{⊕}_{c}exp_{0}^{c}\left(v\right)\right)=\frac{\lambda_{0}^{c}}{\lambda_{x}^{c}}v$$
  ${⊕}_{c}$ is the Möbius addition in $D_{c}^{n}$, $exp_{x}$ is the exponential map at *x*, λ is a smooth function.
Step 2. Embed two sentences using two different hyperbolic RNNs or GRUs. Naturally, a simple RNN can be generalized to the hyperbolic space as follows:
  (14) $$h_{t+1}=\phi^{{⊗}_{c}}\left(W{⊗}_{c}h_{t}{⊕}_{c}U{⊗}_{c}x_{t}{⊕}_{c}b\right),h_{t}\in D_{c}^{n},x_{t}\in D_{c}^{d}$$
  $W\in M_{m,n}\left(R\right)$, U∈$M_{m,d}\left(R\right)$, $b\in D_{c}^{m}$, ϕ is a pointwise nonlinearity, typically tanh, sigmoid, ReLU, etc.
  Under the adjusted GRU architecture, the update gate equation is adjusted as:
  (15) $$h_{t}=h_{t-1}{⊕}_{c}diag\left(z_{t}\right){⊗}_{c}\left(-h_{t-1}{⊕}_{c}{\tilde{h}}_{t}\right)$$
  diag(*x*) denotes the diagonal matrix.
Step 3. Enter sentence embeddings with their hyperbolic or Euclidean squared distances into an FFNN according to their geometry. Here are some of the common operations (Möbius version) defined in the hyperbolic environment:
  As the map from $D_{c}^{n}$ to $D_{c}^{m}$ for f: $R^{n}\to R^{m}$, there the Möbius version of f is defined by:
  (16) $$f^{{⊗}_{c}}\left(x\right):=exp_{0}^{c}\left(f\left(log_{0}^{c}\left(x\right)\right)\right)$$
  Möbius matrix–vector multiplication: If there is a linear map M: $R^{n}\to R^{m}$, identify with its matrix representation, then $\forall x\in D_{c}^{n}$, if Mx≠ 0:
  (17) $$M^{{⊗}_{c}}\left(x\right)=\left(\frac{1}{\sqrt{c}}\right)\tanh\left(\frac{\left||Mx|\right|}{\left||x|\right|}\tanh^{-1}(\sqrt{c}\left||x||\right)\right)\frac{\left||Mx|\right|}{\left||x|\right|}$$
Step 4. Input an MLR (Euclidean or hyperbolic), using the cross-entropy loss on top. To achieve multi-classification, multinomial logistic regression (MLR) (also known as softmax regression) needs to be generalized to Poincaré Ball:
  (18) $$p\left(y=k|x\right)\propto exp\left(sign\left(\langle -p_{k}{⊕}_{c}x,a_{k}\rangle \right)\sqrt{g_{p_{k}}^{c}\left(a_{k},a_{k}\right)}d_{c}\left(x,{\tilde{H}}_{a_{k},p_{k}}^{c}\right)\right),\forall x\in D_{c}^{n}$$
  If there is a K class, k ∈{1,...,K}, $p_{k}\in D_{c}^{n}$, $a_{k}\in T_{pk}D_{c}^{n}$∖{0}

#### 2.2.5. Hyperbolic Graph Convolutions (HGCN)

Hyperbolic graph convolutional neural network (HGCN) [47] is a generalization of inductive GCNs in hyperbolic geometry, which translates GCNs from Euclidean space to hyperbolic geometric space to obtain smaller distortion embeddings. Then, through a hyperbolic attention-based aggregation scheme, the hyperbolic embeddings are finally learned in the graph structure and the concept of node hierarchy in the graph. For HGCN, the steps during aggregating are as follows:

Step 1. Mapping from Euclidean to hyperbolic spaces. The input features are generally Euclidean, and it is necessary to first map the input features to hyperbolic manifolds by exp mapping.
  (19) $$x^{0,H}=exp_{o}^{K}\left(\left(0,x^{0,E}\right)\right)=\left(\sqrt{k}\cosh\left(\frac{\left|\right|x^{0,E}{\left|\right|}_{2}}{\sqrt{k}}\right),\sqrt{k}\sinh\left(\frac{\left|\right|x^{0,E}{\left|\right|}_{2}}{\sqrt{k}}\right)\frac{x^{0,E}}{\left|\right|x^{0,E}{\left|\right|}_{2}}\right)$$
  $x^{0,E}$ is input Euclidean features, $o:=\{\sqrt{K},0,...,0\}$, *o* is the north pole (origin) in $H^{d,K}$, ${\left||v|\right|}_{L}=\sqrt{{\langle v,v\rangle }_{L}}$
Step 2. Multi-stack hyperbolic graph convolution layers. At each layer, the HGCN takes the embeddings of the neighbors in the tangent space of the central node, performs a hyperbolic linear transformation and aggregates them based on attention, and projects the result into a hyperbolic space with a different curvature. That is, in the HGCN layer, it is necessary to pass, if there is a graph G=(γ,ϵ) and Euclidean features ${\left(x^{0,E}\right)}_{i\in \gamma }$:
  (20) $$h_{i}^{\ell ,H}=\left(W^{\ell }{⊗}^{K_{\ell -1}}x_{i}^{\ell -1,H}\right){⊕}_{\ell -1}^{K}b^{\ell }$$
  (21) $$y_{i}^{\ell ,H}=AGG^{K_{\ell -1}}{\left(h^{\ell ,H}\right)}_{i}$$
  (22) $$x_{i}^{\ell ,H}=\sigma^{{⊗}^{K_{\ell -1},K_{\ell }}}\left(y_{i}^{\ell ,H}\right)$$
  where
  (23) $$w_{ij}=SOFTMAX_{j\in N(i)}(MLP(log_{o}^{K}\left(x_{i}^{H}\right)||log_{o}^{K}\left(j_{i}^{H}\right)))$$
  (24) $$AGG^{K}{\left(x^{K}\right)}_{i}=exp_{x_{i}^{H}}^{K}\left(\sum_{j\in N(i)}w_{ij}log_{x_{i}^{H}}^{K}\left(x_{j}^{H}\right)\right)$$
Step 3. Node attributes or links prediction. For link prediction, the probability scores of the edges were calculated using the Fermi–Dirac decoder [58,59] (a generalization of sigmoid):
  (25) $$p\left(\left(i,j\right)\in \epsilon |x_{i}^{L,H},x_{j}^{L,H}\right)={\left[e^{(d_{L}^{K_{L}}{\left(x_{i}^{L,H},x_{j}^{L,H}\right)}^{2}-r)/t}+1\right]}^{-1}$$
  *r* and t are hyperparameters, $d_{L}^{K_{L}}\left(\cdot ,\cdot \right)$ is the hyperbolic distance. The HGCN is then trained by minimizing the cross-entropy loss using negative sampling.
  For node classification, we use logarithmic mapping ${log}_{o}^{K_{L}}\left(\cdot \right)$ to map the output of the last HGCN layer to the tangent space of the origin and then perform Euclidean polynomial logistic regression. Finally, a link prediction regularization objective was added to encourage embedding at the final layer, thus maintaining the structure of the graph.

### 2.3. Experiments

#### 2.3.1. Dataset Settings

We collect a large amount of PPI data from the well-acknowledged STRING database2. Inspired by [39,57], we use the SHS27k and SHS148k datasets in this study and follow the major seven protein–protein interaction types, i.e., including reaction, binding, post-translational modifications (ptm), activation, inhibition, catalysis, and expression. We subsequently extract seven subsets for each dataset and finally obtain fourteen PPI datasets. For each dataset, we have the node features of each protein and confirm the edge connecting different pairs of proteins. The node feature is fixed length that represents the amino acid sequence. The edge is presented as the link between two protein ids. Based on the fourteen datasets, for each dataset, we randomly divide these edges into 85/5/10% for the training dataset, validation dataset, and test dataset.

#### 2.3.2. Implementation Details

All the aforementioned methods are performed with Pytorch [60], Scikit-learn [61], and GraphZoo [47,62]. The parameters that need to be optimized are shown in Table 2. Most of these models share the same set of parameters. Specifically, all models employ two stacked neural network layers, with the first layer reducing the feature dimension from 50 to 16, the second layer maintaining the input feature dimension of 16, and the output feature dimension also being 16. The learning rate is set to 0.01. The weight-decay value is set to 0.001. ReLu is selected as the activation function. A drop-out rate of 0.4 is implemented. Some parameters are different for different models. As the manifold for hyperbolic networks, such as HNN and HGCN, we use the Poincare Ball. As the manifold for graph neural networks, such as GCN and GAT, we employ the Euclidean. GAT has an additional hyperparameter, i.e., the number of heads for multi-head attention, which we set to 4. After 5000 training epochs, all of these models have reached their optimal state.

The operation system we used is Ubuntu 20.04.1 LTS. All of these experiments are conducted on the environment using one AMD EPYC 7502P CPU @ 3.35GHZ and an NVIDIA RTX 3090 card with 24GB of memory. The remaining major library versions are as follows: numpy == 1.23.1, scikit-learn == 1.1.2, torch == 1.12.1, networkx == 2.6.

#### 2.3.3. Evaluation Metrics

For the purpose of assessing the overall effectiveness of these prediction models, we employ two widely used metrics, i.e., average precision (AP) and receiver operating characteristic (ROC), for this classification prediction model performance evaluation. The AP represents a precision–recall curve through a weighted average value of precisions. The computation equation is shown in Equation (26). Here, $Precision_{n}$ and $Recall_{n}$ refer to the precision and recall, respectively. For the ROC, it is a graphical diagram of the classification performance when the discrimination threshold changes. It can be obtained through graphing the proportion of true positives out of the positives, with the fraction of false positives out of the negatives. Overall, a greater AP and ROC value indicates a better prediction performance.

(26) $$AP=\sum_{n}\left(Recall_{n}-Recall_{n-1}\right)\times Precision_{n}$$

## 3. Results and Discussion

We process the raw protein datasets into fourteen subset datasets and conduct comparative studies, respectively. The mentioned models are performed on these datasets. After being fully trained, each graph neural network-based model is used on the testing datasets and outputs the final protein–protein interactions prediction results.

### 3.1. Performance Comparison on SHS27k Dataset

We present the prediction performance comparison between these models in terms of the ROC, AP, and runtime, leveraging the SHS27k dataset. In the following section, we will elaborate on the experimental results.

#### 3.1.1. ROC Comparison on SHS27k Dataset

**Figure 5:**
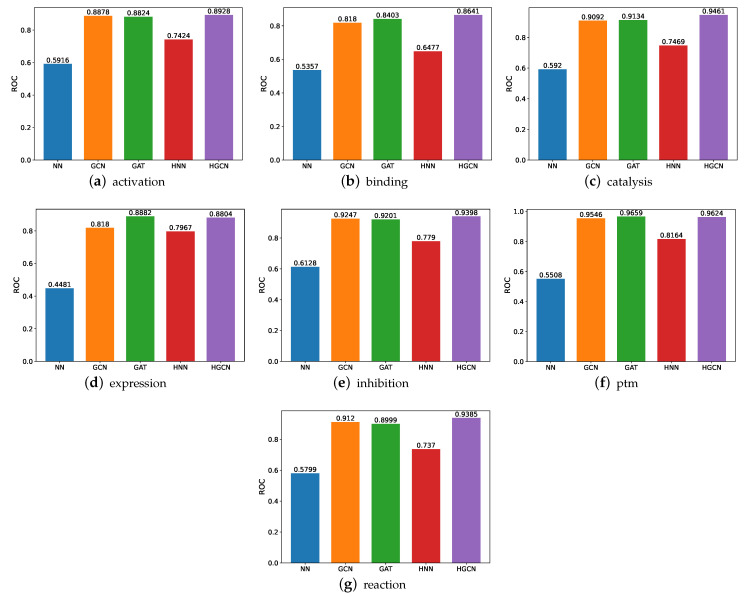
ROC comparison between models for SHS27k.

We demonstrate the comparative study results in terms of the ROC, as shown in Figure 5. The experiments are conducted on the following datasets, including the activation, binding, catalysis, expression, inhibition, post-translational modifications (ptm), and reaction. From these figures, we can observe that the HGCN achieves the best ROC performance compared with the other models in all the SHS27k subsets. This may be caused by the curvature of these datasets. Therefore, the hyperbolic graph neural networks can deal with these situations better. This claim can also be supported by the comparison between the performance by the HNN and NN. As can be shown, the HNN consistently obtains higher ROC results than the NN. In addition, we can also observe that the GCN and GAT models obtain a comparable prediction performance in the ROC, significantly better than the performance of both the traditional neural networks and hyperbolic neural networks. This demonstrates the efficacy of the graph neural networks.

#### 3.1.2. AP Comparison on SHS27k Dataset

**Figure 6:**
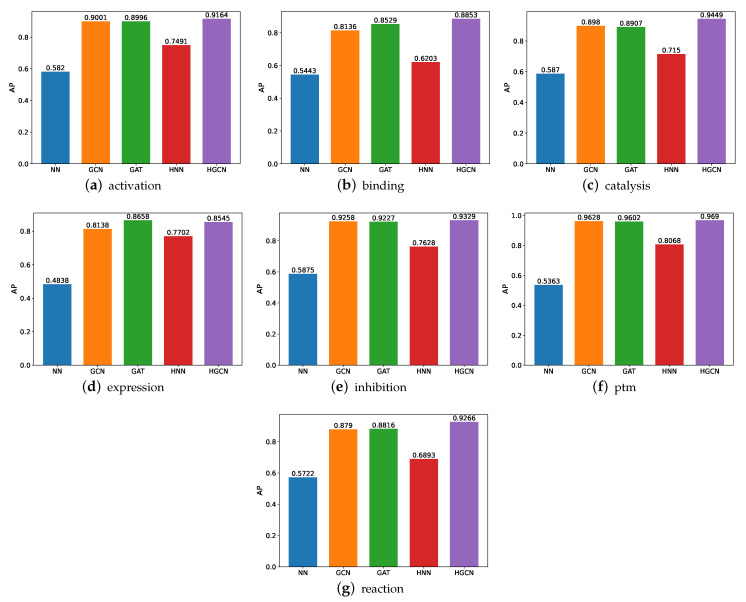
AP comparison between models for SHS27k.

We also conducted comparative experiments on these datasets in terms of the AP and present the experimental results in Figure 6. Based on these results, we can see that the HGCN outperforms the other models in all the SHS27k subsets in regard to the AP performance. Meanwhile, the HNN constantly performs better than the NN. These findings support our aforementioned claims that the hyperbolic networks are particularly well-suited for the protein-related datasets. The graph neural networks also demonstrate their strong capabilities in this field, through the good performance by the GCN and GAT. In addition, we also observe that these models obtain a better performance on the ptm dataset than the other six datasets, while the worst AP performance is observed on the expression dataset.

#### 3.1.3. Runtime Comparison on SHS27k Dataset

**Figure 7:**
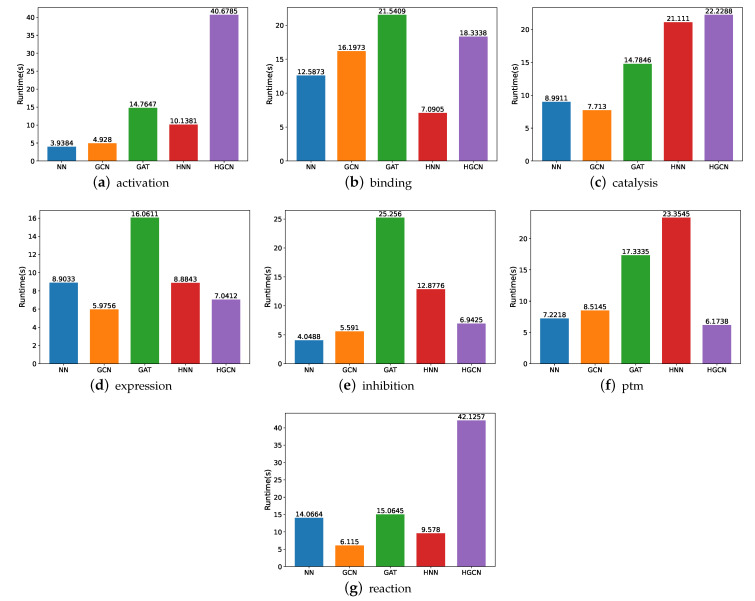
Runtime comparison between models for SHS27k.

In order to gain a better understanding of which one of these models is more effective, we compare their runtime, utilizing various experiments. Figure 7 shows the experimental findings. Here, note that the runtime is calculated in seconds. From these figures, we can observe that the HGCN and GAT models demand more time for the experiment implementation, while the neural networks require the least amount of runtime for the model training and experiments in most of these datasets. The underlying reason may be the feed-forward mechanism which has fewer parameters to be optimized.

### 3.2. Prediction Comparison on SHS148k Dataset

To verify the robustness of these models, we further conduct experiments on another dataset, i.e., SHS148k. The prediction performance comparison among these models with regard to the ROC, AP, and runtime will be described in the following section.

#### 3.2.1. ROC Comparison on SHS148k Dataset

**Figure 8:**
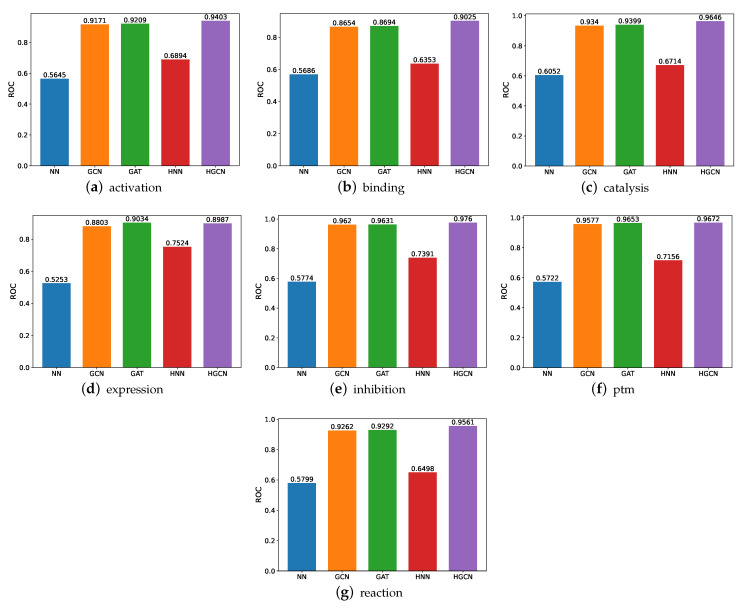
ROC comparison between models for SHS148k.

The ROC comparison performance on the SHS148k dataset is demonstrated in Figure 8. From these figures, we can observe the following findings. First, similar to the performance on the SHS27k dataset, the HGCN model achieves the best ROC performance compared with the other models, and the hyperbolic neural network outperforms the traditional three-layer neural network on the seven subsets. This provides consistent support for our previous claims that the protein–protein interactions datasets are more suitable to be modeled in the hyperbolic space rather than the Euclidean space. Second, the HGCN achieves the better prediction performance on the SHS148k dataset than the SHS27k dataset, including all the seven subsets. The reason behind this may be the larger amount of data in SHS148k, which can help these neural networks-based models demonstrate better learning capabilities.

#### 3.2.2. AP Comparison on SHS148k Dataset

**Figure 9:**
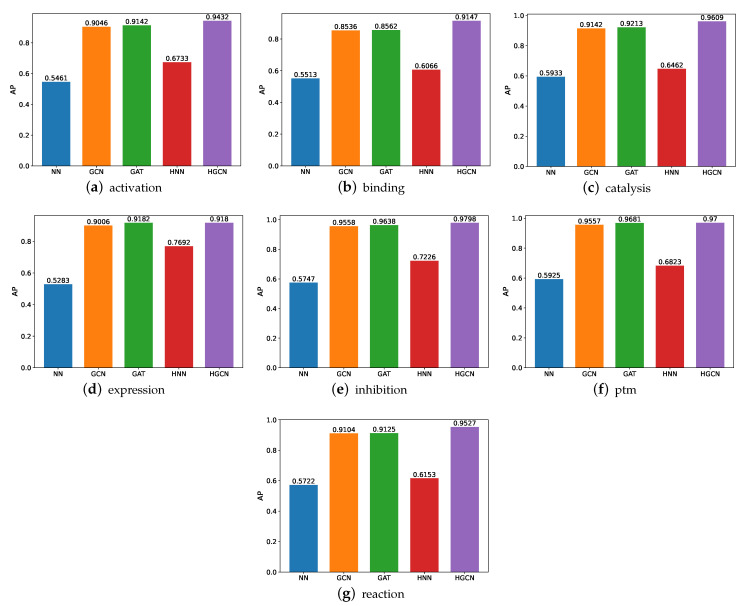
AP comparison between models for SHS148k.

The AP comparison performance is also carried out, and the experimental results are shown in Figure 9. On the basis of these results, we can see that, similar to the performance on SHS27k, the HGCN gains the best performance compared with the other models on all the SHS148k subsets in regard to the AP metric. In addition, we observe that the HGCN can achieve a comparable prediction performance on the activation, catalysis, inhibition, ptm, and reaction subsets of SHS148k. Meanwhile, for the SHS27k dataset, this model obtains a comparable performance on the catalysis, inhibition, and ptm subsets. It is possible that the larger dataset helps bring a better prediction performance.

#### 3.2.3. Runtime Comparison on SHS148k Dataset

**Figure 10:**
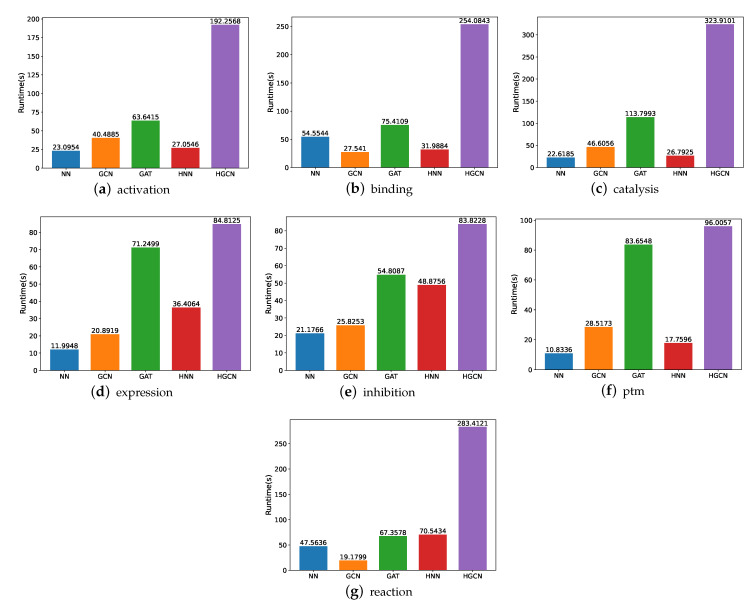
Runtime comparison between models for SHS148k.

For the SHS148k dataset, we also carry out the runtime performance comparison, and the results are demonstrated in Figure 10. Based on these figures, we are able to deduce that the execution of these models takes a significantly longer amount of time. For example, for the experiments on the activation subsets, the HGCN demands 7 times the runtime on SHS148k than on SHS27k. For the catalysis subset, the HGCN method needs a runtime of 323 s on the SHS148k dataset. This is over 15 times longer than the runtime required for the SHS27k dataset, which requires only 22 s for the experiments to be carried out.

## 4. Conclusions

The study of the protein–protein interaction is of critical importance for further investigating protein function, gene regulation, immune recognition, disease mechanism, and drug design. In this paper, we conducted a comparative study for protein–protein interaction prediction using multiple graph neural network-based models, including the GCN, GAT, HNN, and HGCN. The performances of the different graph neural networks for PPI prediction are compared using fourteen datasets. The experimental results demonstrate that graph neural network-based models are powerful for solving the protein–protein interaction prediction tasks, no matter the size of these PPI datasets, which are highly diverse, with the number of protein interactions ranging from 1572 to 102,964. This indicates the stability of the GNN model in solving the PPI problem; the HGCN generally outperforms the other types of graph neural networks on all the datasets. It shows the promising potential of the different graph neural networks for predicting protein–protein interactions.

There are still some limitations despite the thorough research results we obtained. First, PPI is usually modeled as a link prediction task in graphs using GNNs. For PPI prediction, most existing approaches use generic graph representation learners. Thus, one open future task is to design graph models that focus on link prediction to solve PPI problems, which is a promising idea. Second, the environment in which a protein resides, such as different species or body tissues, has unique characteristics, which can be modeled as global information in the graph. One of the future works will be combining global information to perform the link prediction on graphs.

## Figures and Tables

**Table 1:** Dataset Description.

Datasets	Interaction Type	# PPI	Positive Samples	Datasets	Interaction Type	# PPI	Positive Samples
SHS27k	reaction	18,162	1.09%	SHS148k	reaction	102,964	0.67%
	binding	16,056	0.47%		binding	93,632	0.28%
	ptm	2872	1.05%		ptm	20,154	0.38%
	activation	7400	0.40%		activation	42,516	0.23%
	inhibition	5550	1.48%		inhibition	34,712	0.76%
	catalysis	11,796	0.79%		catalysis	67,168	0.50%
	expression	1572	0.40%		expression	7896	0.16%

**Table 2:** Number of parameters of different models.

Model	# Parameters
NN	1088
GCN	1088
GAT	1120
HNN	1088
HGCN	1088

## Data Availability

All the code and datasets used in this study can be found in this online repository https://github.com/ZJUDataIntelligence/GNN-PPI) (accessed on 15 August 2022).

## Abbreviations

The following abbreviations are used in this manuscript:

PPI	Protein–Protein Interactions
NN	Neural Networks
GCN	Graph Convolutional Neural Networks
GAT	Graph Attention Networks
HNN	Hyperbolic Neural Networks
HGCN	Hyperbolic Graph Convolutions
